# ECDC publishes 2015 surveillance data on antimicrobial resistance and antimicrobial consumption in Europe

**DOI:** 10.2807/1560-7917.ES.2016.21.46.30399

**Published:** 2016-11-17

**Authors:** K Weist, L Diaz Högberg

**Affiliations:** 1 European Centre for Disease Prevention and Control (ECDC), Stockholm, Sweden

**Keywords:** European Antibiotic Awareness Day

On the occasion of the European Antibiotic Awareness Day on 18 November 2016, the European Centre for Disease Prevention and Control will release 2015 data on antimicrobial resistance [1] and antimicrobial consumption [2] from 30 European Union (EU) and European Economic Area (EEA) countries. The data are accompanied by summaries highlighting the latest trends [3,4].

The latest data from the European Antimicrobial Resistance Surveillance Network (EARS-Net) show high and increasing resistance percentages for Gram-negative bacteria in many parts of Europe. This is reflected by the increase in combined resistance to third-generation cephalosporins, fluoroquinolones and aminoglycosides resistance at EU/EEA level between 2012 and 2015 for both *Escherichia coli* and *Klebsiella pneumoniae*. Although carbapenem resistance percentages remained low for most countries in 2015, resistance to carbapenems increased significantly for *K. pneumoniae* at EU/EEA level over the last four years. Data on polymyxin resistance in EARS-Net are sparse, but some countries, especially those with high percentages of carbapenem resistance, report presence of isolates with polymyxin resistance.

The latest data from the European Surveillance of Antimicrobial Consumption Network (ESAC-Net) showed that overall consumption of antibiotics in the community remained unchanged from 2011 to 2015. However, when measuring the antibiotic consumption as a number of packages per 1,000 inhabitants and per day (used by ESAC-Net as the best available surrogate for prescriptions), six countries experienced a significant decrease during the period the same period.

In the hospital sector, the overall consumption of antibiotics remained stable in the EU/EEA. At the national level, antibiotic consumption of carbapenems and polymyxins used to treat patients with serious multidrug-resistant bacteria is still at a low level compared to the overall consumption of antibiotics for systemic use in the hospital sector. However, significant increasing trends in the consumption of carbapenems (six countries) and polymyxins (eight countries) were reported for the period 2011‒2015.

In countries with high levels of multidrug resistance, including resistance to carbapenems, only a few therapeutic options are available; among these are polymyxins (e.g. colistin). The presence of isolates with resistance to polymyxins and increasing trends in polymyxin consumption in several countries is an important warning that options for the treatment of infected patients are becoming even more limited.
